# 4-(4-Oxopent-2-en-2-yl­amino)-1,2,4-triazol-1-ium-5-thiol­ate

**DOI:** 10.1107/S1600536809019850

**Published:** 2009-06-06

**Authors:** Xing-Cheng Zhu, Qi-Long Zhang, Yun-Qian Zhang, Bi-Xue Zhu

**Affiliations:** a Key Laboratory of Macrocyclic and Supramolecular Chemistry of Guizhou Province, Guizhou University, Guiyang 550025, People’s Republic of China

## Abstract

In the title compound, C_8_H_12_N_4_OS, an intra­molecular N—H⋯O hydrogen bond links the imine N atom to the oxo O atom. In the crystal, mol­ecules are linked by inter­molecular N—H⋯O and N—H⋯S hydrogen bonds, forming a two-dimensional framework.

## Related literature

For Schiff base metal complexes, see: Lacroix (2001 ▶); Sabater *et al.* (2001 ▶). For the use of 1,2,4-triazole and its derivatives as ligands to bridge metal ions, see: Yi *et al.* (2004 ▶).
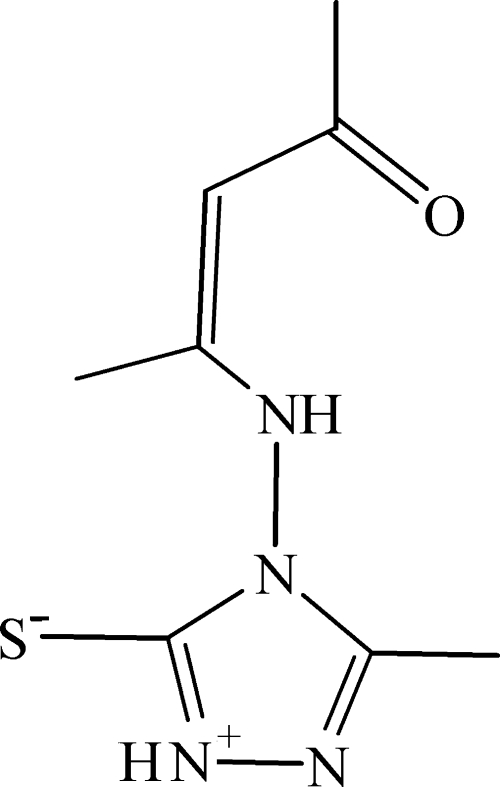


## Experimental

### 

#### Crystal data

C_8_H_12_N_4_OS
*M* *_r_* = 212.28
Monoclinic, 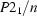

*a* = 10.620 (6) Å
*b* = 9.520 (5) Å
*c* = 10.764 (5) Å
β = 99.560 (14)°
*V* = 1073.2 (10) Å^3^
*Z* = 4
Mo *K*α radiation
μ = 0.28 mm^−1^
*T* = 293 K
0.26 × 0.23 × 0.18 mm

#### Data collection

Bruker SMART APEXII CCD area-detector diffractometer
Absorption correction: multi-scan (*SADABS*; Bruker, 2005 ▶) *T*_min_ = 0.932, *T*_max_ = 0.952
9199 measured reflections
2071 independent reflections
1688 reflections with *I* > 2σ(*I*)
*R*_int_ = 0.030

#### Refinement

*R*[*F* ^2^ > 2σ(*F* ^2^)] = 0.038
*wR*(*F* ^2^) = 0.105
*S* = 1.06
2071 reflections
130 parameters
H-atom parameters constrained
Δρ_max_ = 0.24 e Å^−3^
Δρ_min_ = −0.22 e Å^−3^

### 

Data collection: *APEX2* (Bruker, 2005 ▶); cell refinement: *SAINT* (Bruker, 2005 ▶); data reduction: *SAINT*; program(s) used to solve structure: *SHELXS97* (Sheldrick, 2008 ▶); program(s) used to refine structure: *SHELXL97* (Sheldrick, 2008 ▶); molecular graphics: *ORTEP-3 for Windows* (Farrugia, 1997 ▶); software used to prepare material for publication: *WinGX* (Farrugia, 1999 ▶).

## Supplementary Material

Crystal structure: contains datablocks global, I. DOI: 10.1107/S1600536809019850/at2792sup1.cif

Structure factors: contains datablocks I. DOI: 10.1107/S1600536809019850/at2792Isup2.hkl

Additional supplementary materials:  crystallographic information; 3D view; checkCIF report

## Figures and Tables

**Table 1:** Hydrogen-bond geometry (Å, °)

*D*—H⋯*A*	*D*—H	H⋯*A*	*D*⋯*A*	*D*—H⋯*A*
N1—H1⋯O1	0.86	2.03	2.659 (2)	130
N1—H1⋯S1^i^	0.86	2.81	3.4127 (19)	129
N3—H3*A*⋯O1^ii^	0.86	1.93	2.772 (2)	166

Symmetry codes: (i) 
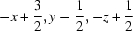
; (ii) 
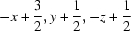
.

**Fig. 1.**
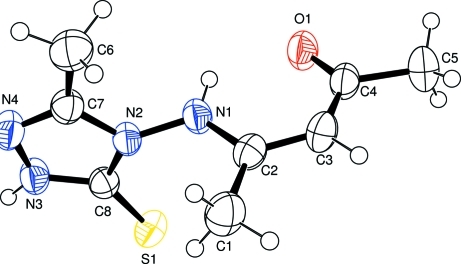
The molecular structure of (I) showing the atom-labelling scheme. Displacement ellipsoids are drawn at the 50% probability level.

## supplementary crystallographic information

### Comment

Schiff base metal complexes have been widely investigated for their properties and applications in different fields, catalysis (Sabater *et al.*, 2001), materials chemistry (Lacroix 2001) and simple organic molecules, such as 1,2,4-triazole and its derivatives, which usually studied as precursors of compounds with importance in medicine biology and industry, have gained more and more interest as ligands to bridge metal ions due to their potential bridging fashions (Yi *et al.*, 2004). In this work, we report a crystal structure of 3-methyl-4-amino-5-mercapto-1,2,4- triazole, (I).

The crystal structure of the title compound is shown in Fig. 1. The molecule is a non-coplanar structure, an intramolecular N1—H1···O1 hydrogen bonds linking the amines N1 atoms to the enolic O1 atoms. As shown in Fig. 2, the molecules of the title compound are lined up by the intermolecular N1—H1···S1 and N3—H3A···O1 interactions (Table 2) forming a two-dimensional framework.

### Experimental

Acetylacetone (1.0 g, 10 mmol) was added to an ethanol solution containing 3-methyl-4-amino-5-mercapto-1,2,4-triazole (1.3 g, 10 mmol). The mixture was heated, stirring for 24 h. The yellow residue yielded and was removed from the solution by filtration, washing with ethanol 3 times, 1.47 g, in a yield of 69%. Single crystals suitable for X-ray diffraction were obtained from an ethanol-CH_2_Cl_2_ mixture (1:1, v:v) by slow evaporation at room temperature.

### Refinement

All H atoms were placed in calculated positions and refined as riding, with C—H = 0.93–0.96 Å, N—H = 0.8600 Å, and *U*_iso_(H) = 1.2–1.5*U*_eq_(C, N).

### Crystal data

**Table:**

C_8_H_12_N_4_OS	*F*(000) = 448
*M**_r_* = 212.28	*D*_x_ = 1.314 Mg m^−^^3^
Monoclinic, *P*2_1_/*n*	Mo *K*α radiation, λ = 0.71073 Å
Hall symbol: -P 2yn	Cell parameters from 9199 reflections
*a* = 10.620 (6) Å	θ = 2.5–26.0°
*b* = 9.520 (5) Å	µ = 0.28 mm^−^^1^
*c* = 10.764 (5) Å	*T* = 293 K
β = 99.560 (14)°	Prism, yellow
*V* = 1073.2 (10) Å^3^	0.26 × 0.23 × 0.18 mm
*Z* = 4	

### Data collection

**Table:**

Bruker SMART APEXII CCD area-detector diffractometer	2071 independent reflections
Radiation source: fine-focus sealed tube	1688 reflections with *I* > 2σ(*I*)
graphite	*R*_int_ = 0.030
φ and ω scans	θ_max_ = 26.0°, θ_min_ = 2.5°
Absorption correction: multi-scan (*SADABS*; Bruker, 2005)	*h* = −12→13
*T*_min_ = 0.932, *T*_max_ = 0.952	*k* = −11→11
9199 measured reflections	*l* = −13→13

### Refinement

**Table:**

Refinement on *F*^2^	Primary atom site location: structure-invariant direct methods
Least-squares matrix: full	Secondary atom site location: difference Fourier map
*R*[*F*^2^ > 2σ(*F*^2^)] = 0.038	Hydrogen site location: inferred from neighbouring sites
*wR*(*F*^2^) = 0.105	H-atom parameters constrained
*S* = 1.06	*w* = 1/[σ^2^(*F*_o_^2^) + (0.0494*P*)^2^ + 0.3337*P*] where *P* = (*F*_o_^2^ + 2*F*_c_^2^)/3
2071 reflections	(Δ/σ)_max_ < 0.001
130 parameters	Δρ_max_ = 0.24 e Å^−^^3^
0 restraints	Δρ_min_ = −0.22 e Å^−^^3^

### Special details

**Table:**

Geometry. All e.s.d.'s (except the e.s.d. in the dihedral angle between two l.s. planes) are estimated using the full covariance matrix. The cell e.s.d.'s are taken into account individually in the estimation of e.s.d.'s in distances, angles and torsion angles; correlations between e.s.d.'s in cell parameters are only used when they are defined by crystal symmetry. An approximate (isotropic) treatment of cell e.s.d.'s is used for estimating e.s.d.'s involving l.s. planes.
Refinement. Refinement of *F*^2^ against ALL reflections. The weighted *R*-factor *wR* and goodness of fit *S* are based on *F*^2^, conventional *R*-factors *R* are based on *F*, with *F* set to zero for negative *F*^2^. The threshold expression of *F*^2^ > σ(*F*^2^) is used only for calculating *R*-factors(gt) *etc*. and is not relevant to the choice of reflections for refinement. *R*-factors based on *F*^2^ are statistically about twice as large as those based on *F*, and *R*- factors based on ALL data will be even larger.

### Fractional atomic coordinates and isotropic or equivalent isotropic displacement parameters (Å^2^)

**Table:**

	*x*	*y*	*z*	*U*_iso_*/*U*_eq_	
C1	0.6978 (2)	0.4476 (2)	0.6177 (2)	0.0581 (6)	
H1A	0.6385	0.4804	0.6695	0.087*	
H1B	0.7230	0.5246	0.5697	0.087*	
H1C	0.7717	0.4092	0.6702	0.087*	
C2	0.63580 (17)	0.3365 (2)	0.53014 (16)	0.0413 (4)	
C3	0.51805 (18)	0.2824 (2)	0.53712 (17)	0.0474 (5)	
H3	0.4781	0.3133	0.6027	0.057*	
C4	0.45166 (17)	0.1828 (2)	0.45229 (17)	0.0437 (4)	
C5	0.31956 (19)	0.1390 (3)	0.4704 (2)	0.0598 (6)	
H5A	0.2574	0.1966	0.4191	0.090*	
H5B	0.3113	0.1500	0.5573	0.090*	
H5C	0.3058	0.0423	0.4464	0.090*	
C6	0.9660 (2)	0.1873 (2)	0.5572 (2)	0.0579 (5)	
H6A	1.0551	0.1644	0.5677	0.087*	
H6B	0.9162	0.1048	0.5318	0.087*	
H6C	0.9457	0.2212	0.6355	0.087*	
C7	0.93642 (17)	0.29676 (19)	0.45994 (16)	0.0420 (4)	
C8	0.81958 (17)	0.45095 (18)	0.33090 (15)	0.0377 (4)	
N1	0.70361 (13)	0.28695 (16)	0.44362 (13)	0.0430 (4)	
H1	0.6761	0.2136	0.4010	0.052*	
N2	0.81498 (13)	0.34888 (15)	0.42069 (13)	0.0381 (3)	
N3	0.94393 (14)	0.45391 (16)	0.32250 (14)	0.0445 (4)	
H3A	0.9753	0.5092	0.2723	0.053*	
N4	1.01765 (14)	0.35996 (17)	0.40183 (15)	0.0483 (4)	
O1	0.49708 (12)	0.13376 (15)	0.36199 (13)	0.0536 (4)	
S1	0.69902 (5)	0.54628 (5)	0.25545 (4)	0.04948 (19)	

### Atomic displacement parameters (Å^2^)

**Table:**

	*U*^11^	*U*^22^	*U*^33^	*U*^12^	*U*^13^	*U*^23^
C1	0.0537 (13)	0.0681 (14)	0.0536 (11)	−0.0014 (10)	0.0118 (10)	−0.0165 (10)
C2	0.0402 (10)	0.0460 (10)	0.0389 (9)	0.0044 (8)	0.0103 (8)	0.0013 (7)
C3	0.0418 (11)	0.0602 (12)	0.0439 (10)	0.0030 (9)	0.0180 (8)	−0.0033 (9)
C4	0.0370 (10)	0.0495 (11)	0.0474 (10)	0.0029 (8)	0.0151 (8)	0.0062 (8)
C5	0.0426 (12)	0.0754 (15)	0.0653 (13)	−0.0085 (10)	0.0198 (10)	0.0014 (11)
C6	0.0566 (13)	0.0560 (12)	0.0597 (12)	0.0071 (10)	0.0055 (10)	0.0110 (10)
C7	0.0369 (10)	0.0440 (10)	0.0447 (9)	0.0003 (8)	0.0055 (8)	−0.0035 (8)
C8	0.0379 (10)	0.0402 (9)	0.0360 (8)	−0.0018 (7)	0.0089 (7)	−0.0040 (7)
N1	0.0388 (9)	0.0446 (9)	0.0490 (8)	−0.0086 (7)	0.0175 (7)	−0.0082 (7)
N2	0.0316 (8)	0.0411 (8)	0.0427 (8)	−0.0030 (6)	0.0092 (6)	−0.0012 (6)
N3	0.0359 (9)	0.0500 (9)	0.0493 (9)	−0.0028 (7)	0.0116 (7)	0.0062 (7)
N4	0.0339 (8)	0.0570 (10)	0.0536 (9)	0.0008 (7)	0.0059 (7)	0.0037 (7)
O1	0.0461 (8)	0.0604 (9)	0.0591 (8)	−0.0084 (6)	0.0223 (7)	−0.0143 (7)
S1	0.0430 (3)	0.0588 (3)	0.0476 (3)	0.0104 (2)	0.0103 (2)	0.0053 (2)

### Geometric parameters (Å, °)

**Table:**

C1—C2	1.495 (3)	C6—C7	1.474 (3)
C1—H1A	0.9600	C6—H6A	0.9600
C1—H1B	0.9600	C6—H6B	0.9600
C1—H1C	0.9600	C6—H6C	0.9600
C2—N1	1.353 (2)	C7—N4	1.295 (2)
C2—C3	1.366 (3)	C7—N2	1.381 (2)
C3—C4	1.420 (3)	C8—N3	1.339 (2)
C3—H3	0.9300	C8—N2	1.377 (2)
C4—O1	1.245 (2)	C8—S1	1.6664 (19)
C4—C5	1.507 (3)	N1—N2	1.380 (2)
C5—H5A	0.9600	N1—H1	0.8600
C5—H5B	0.9600	N3—N4	1.386 (2)
C5—H5C	0.9600	N3—H3A	0.8600
			
C2—C1—H1A	109.5	C7—C6—H6B	109.5
C2—C1—H1B	109.5	H6A—C6—H6B	109.5
H1A—C1—H1B	109.5	C7—C6—H6C	109.5
C2—C1—H1C	109.5	H6A—C6—H6C	109.5
H1A—C1—H1C	109.5	H6B—C6—H6C	109.5
H1B—C1—H1C	109.5	N4—C7—N2	110.40 (16)
N1—C2—C3	120.20 (17)	N4—C7—C6	126.26 (17)
N1—C2—C1	116.86 (17)	N2—C7—C6	123.32 (16)
C3—C2—C1	122.92 (17)	N3—C8—N2	102.27 (15)
C2—C3—C4	125.30 (16)	N3—C8—S1	129.95 (14)
C2—C3—H3	117.4	N2—C8—S1	127.77 (14)
C4—C3—H3	117.4	C2—N1—N2	122.95 (15)
O1—C4—C3	122.48 (17)	C2—N1—H1	118.5
O1—C4—C5	119.12 (18)	N2—N1—H1	118.5
C3—C4—C5	118.38 (17)	C8—N2—N1	123.94 (14)
C4—C5—H5A	109.5	C8—N2—C7	109.16 (14)
C4—C5—H5B	109.5	N1—N2—C7	125.19 (15)
H5A—C5—H5B	109.5	C8—N3—N4	114.05 (15)
C4—C5—H5C	109.5	C8—N3—H3A	123.0
H5A—C5—H5C	109.5	N4—N3—H3A	123.0
H5B—C5—H5C	109.5	C7—N4—N3	104.12 (15)
C7—C6—H6A	109.5		
			
N1—C2—C3—C4	−5.7 (3)	C2—N1—N2—C7	105.0 (2)
C1—C2—C3—C4	176.25 (19)	N4—C7—N2—C8	0.6 (2)
C2—C3—C4—O1	0.7 (3)	C6—C7—N2—C8	179.02 (17)
C2—C3—C4—C5	−177.3 (2)	N4—C7—N2—N1	166.12 (16)
C3—C2—N1—N2	171.33 (16)	C6—C7—N2—N1	−15.5 (3)
C1—C2—N1—N2	−10.5 (3)	N2—C8—N3—N4	−0.11 (19)
N3—C8—N2—N1	−166.02 (14)	S1—C8—N3—N4	179.01 (13)
S1—C8—N2—N1	14.8 (2)	N2—C7—N4—N3	−0.62 (19)
N3—C8—N2—C7	−0.27 (18)	C6—C7—N4—N3	−178.99 (18)
S1—C8—N2—C7	−179.42 (13)	C8—N3—N4—C7	0.5 (2)
C2—N1—N2—C8	−91.5 (2)		

### Hydrogen-bond geometry (Å, °)

**Table:**

*D*—H···*A*	*D*—H	H···*A*	*D*···*A*	*D*—H···*A*
N1—H1···O1	0.86	2.03	2.659 (2)	130
N1—H1···S1^i^	0.86	2.81	3.4127 (19)	129
N3—H3A···O1^ii^	0.86	1.93	2.772 (2)	166

Symmetry codes: (i) −*x*+3/2, *y*−1/2, −*z*+1/2; (ii) −*x*+3/2, *y*+1/2, −*z*+1/2.
